# Selenium deficiency induces spleen pathological changes in pigs by decreasing selenoprotein expression, evoking oxidative stress, and activating inflammation and apoptosis

**DOI:** 10.1186/s40104-021-00587-x

**Published:** 2021-05-17

**Authors:** Shuang Li, Wenjuan Sun, Kai Zhang, Jiawei Zhu, Xueting Jia, Xiaoqing Guo, Qingyu Zhao, Chaohua Tang, Jingdong Yin, Junmin Zhang

**Affiliations:** 1 State Key Laboratory of Animal Nutrition, Institute of Animal Sciences of Chinese Academy of Agricultural Sciences, Beijing, 100193 China; 2 State Key Laboratory of Animal Nutrition, College of Animal Science and Technology, China Agricultural University, Beijing, 100193 China; 3 Scientific Observing and Experiment Station of Animal Genetic Resources and Nutrition in North China of Ministry of Agriculture and Rural Affairs, Institute of Animal Sciences of Chinese Academy of Agricultural Sciences, Beijing, 100193 China

**Keywords:** Apoptosis, Inflammation, Oxidative stress, Pigs, Selenium deficiency, Spleen

## Abstract

**Background:**

The immune system is one aspect of health that is affected by dietary selenium (Se) levels and selenoprotein expression. Spleen is an important immune organ of the body, which is directly involved in cellular immunity. However, there are limited reports on Se levels and spleen health. Therefore, this study established a Se-deficient pig model to investigate the mechanism of Se deficiency-induced splenic pathogenesis.

**Methods:**

Twenty-four pure line castrated male Yorkshire pigs (45 days old, 12.50 ± 1.32 kg, 12 full-sibling pairs) were divided into two equal groups and fed Se-deficient diet (0.007 mg Se/kg) or Se-adequate diet (0.3 mg Se/kg) for 16 weeks. At the end of the trial, blood and spleen were collected to assay for erythroid parameters, the osmotic fragility of erythrocytes, the spleen index, histology, terminal deoxynucleotidyl transferase nick-end labeling (TUNEL) staining, Se concentrations, the selenogenome, redox status, and signaling related inflammation and apoptosis.

**Results:**

Dietary Se deficiency decreased the erythroid parameters and increased the number of osmotically fragile erythrocytes (*P* < 0.05). The spleen index did not change, but hematoxylin and eosin and TUNEL staining indicated that the white pulp decreased, the red pulp increased, and splenocyte apoptosis occurred in the Se deficient group. Se deficiency decreased the Se concentration and selenoprotein expression in the spleen (*P* < 0.05), blocked the glutathione and thioredoxin antioxidant systems, and led to redox imbalance. Se deficiency activated the NF-κB and HIF-1α transcription factors, thus increasing pro-inflammatory cytokines (IL-1β, IL-6, IL-8, IL-17, and TNF-α), decreasing anti-inflammatory cytokines (IL-10, IL-13, and TGF-β) and increasing expression of the downstream genes *COX-2* and *iNOS* (*P* < 0.05), which in turn induced inflammation. In addition, Se-deficiency induced apoptosis through the mitochondrial pathway, upregulated apoptotic genes (*Caspase3*, *Caspase8*, and *Bak*), and downregulated antiapoptotic genes (*Bcl-2*) (*P* < 0.05) at the mRNA level, thus verifying the results of TUNEL staining.

**Conclusions:**

These results indicated that Se deficiency induces spleen injury through the regulation of selenoproteins, oxidative stress, inflammation and apoptosis.

**Supplementary Information:**

The online version contains supplementary material available at 10.1186/s40104-021-00587-x.

## Introduction

Selenium (Se) is a trace element essential for life activities, with important functions in the antioxidant defense system, thyroid hormone metabolism, and immune system [1, 2]. The spleen and lymph nodes together constitute most of the peripheral immune tissue, and several studies have shown that the spleen is sensitive to the Se supply. Se deficiency leads to severe oxidative stress and inflammation of the spleen, thus impairing immune function [3, 4]. Similarly, feeding of a high-Se diet also inhibits immune responses and increases oxidative stress in chicken and fish splenocytes [5–7]. A balanced dietary Se supply is highly important for the spleen’s normal immune function.

Oxidative stress occurs as a result of increased reactive oxygen species (ROS) production and/or an impaired antioxidant defense system. Se deficiency leads to oxidative stress primarily by impairing the two major antioxidant systems of glutathione peroxidase (GPX) and thioredoxin reductase (TXNRD), and decreasing the expression of other antioxidant selenoproteins [8, 9]. Depending on the concentration of ROS, different ROS-sensitive transcription factors are activated, including nuclear factor κB (NF-κB), nuclear erythroid-2 related factors (Nrf2), hypoxia inducible factor-1 alpha (HIF-1α), β-catenin/Wnt, activator protein-1 (AP-1), p53, and peroxisome proliferator activated receptor-γ [10, 11]. Intermediate levels of ROS activate the AP-1 transcription factor and NF-κB signaling pathway, thus triggering inflammatory processes [12]. NF-κB regulates the inflammatory process by regulating the transcription of cytokines, chemokines, cell adhesion molecules, complement cascades and acute phase proteins [13]. AP-1 is a proinflammatory element that controls the expression of cytokines and matrix-degrading matrix metalloproteases at the mRNA synthesis level by directly binding their promoter AP-1 binding motifs [14]. HIF-1α also plays a role in the synthesis of pro-inflammatory cytokines such as interleukin-1 beta (IL-1β) [15]. Oxidative stress has an important role in the activation of the NOD-like receptor protein 3 inflammasome [16]. High level of oxidative stress and inflammation are inseparably linked, as each begets and amplifies the other.

Excess cellular levels of ROS cause damage to proteins, nucleic acids, lipids, membranes and organelles such as mitochondria, thereby leading to activation of cell death processes such as apoptosis. Oxidative stress regulates apoptosis in a manner mainly mediated by mitochondria, death receptors and the endoplasmic reticulum [17]. Several studies have shown that spleen tissue is sensitive to oxidative stress caused by various factors, such as viruses, mycotoxins and heavy metals, thus further leading to splenocyte apoptosis [18–20]. In addition, dietary Se deficiency promotes oxidative stress, inflammation and apoptosis, and aggravates the spleen immunotoxicity induced by aflatoxin B_1_ [21]. In chickens, Se deficiency-induced oxidative stress and nitric oxide cause apoptosis in immune tissues, including the spleen, thymus and bursa of Fabricius [22]. In other species, the mechanism of Se deficiency-related damage in the spleen is less reported. Therefore, we created a model using a pure line of pigs to explore the spleen damage pathway caused by Se deficiency, as well as the possible underlying molecular mechanism. Because the organ size, physiology and selenoprotein profile of pigs are similar to those of humans [23], this research might potentially provide basic data for investigating human nutrition and health problems.

## Materials and methods

### Animals, treatments and sample collection

All animal experiments were approved by the Animal Care and Use Committee of the Institute of Animal Science, Chinese Academy of Agricultural Sciences. A total of 24 pure line castrated male Yorkshire pigs (45 days old, 12 full-sibling pairs) were divided into two equal groups, housed in a climate controlled facility (20–25 °C) in ventilated cages (one pig per cage), and given ad libitum access to feed and water. One group was fed a basal diet (Supplemental Table 1) composed of corn and soybeans obtained from a Se-deficient region, HeiLongJiang, China (Se-deficient group, Se-D); the final Se content in the diet was 0.007 mg/kg, as determined by inductively coupled plasma mass spectrometry. The other group was fed a basal diet supplemented with selenomethionine (SeMet, purity of 98%, J&K Chemical, Shanghai, China) (Se-adequate group, Se-A). The dietary Se concentration in the Se-A group was 0.3 mg/kg, the value recommended by feeding standard of swine (China, NY/T 65–2004 [24]). The trial lasted 16 weeks, and at the end of the experiment, immediately after the 12 h fasting period all pigs were sampled for blood and were slaughtered. Blood samples were collected from the precaval vein and placed in heparin sodium and ethylene diamine tetra acetic acid tubes, and then stored at 4 °C until analyses of erythroid parameters and osmotic fragility of erythrocytes (complete the determination within 4 h). All pigs were slaughtered through electric shock and exsanguinated, and the spleens were rapidly removed and washed with ice-cold isotonic saline. The spleen tissues were divided; one part (5 mm × 5 mm × 5 mm) was fixed in 4% paraformaldehyde for histologic examination, and the remaining part was minced with surgical scissors, snap-frozen in liquid nitrogen and stored at − 80 °C.

### Erythroid parameters and analysis of osmotic fragility of erythrocytes

Blood was analyzed for red blood cells (RBC), hemoglobin (HGB), hematocrit (HCT), mean corpuscular volume (MCV), mean corpuscular hemoglobin (MCH), and mean corpuscular hemoglobin concentration (MCHC) with a Mindray BC-2800Vet auto hematology analyzer (Shenzhen Mindray Bio-Medical Electronics Co., Ltd., Shenzhen, China). Determination of the osmotic fragility of erythrocytes was performed according to a previously described method [25]. Heparinized venous blood (50 μL) was added into tubes with 5 mL of buffered salt solution with increasing concentrations of NaCl (NaCl 0, 0.10%, 0.20%, 0.30%, 0.40%, 0.50%, 0.60%, 0.70%, 0.80%, and 0.90%). The tubes were gently mixed and incubated at 25 °C for 30 min. The samples were centrifuged at 3,000 × *g* for 5 min, and 200 μL of supernatant was evaluated spectrophotometrically at 540 nm. The percentage of hemolyzed erythrocytes was obtained by comparison of the absorbance of samples in NaCl-phosphate buffer (hypotonic stress buffer) with that in distilled water (100% hemolysis).

### Spleen index and se concentrations analysis

The spleen index was calculated according to the following formula: spleen index (g/kg) = weight of spleen/body weight. The concentrations of Se in the spleen were measured with Agilent 7900 inductively coupled plasma mass spectrometry (Agilent Technologies, Santa Clara, CA, USA) as previously described [26]. Certified reference materials (pig liver, GBW10051; chicken muscle, GBW10018) were used for quality control in Se detection.

### Histologic and terminal deoxynucleotidyl transferase nick-end labeling (TUNEL) analysis

The spleen tissues were fixed in 4% paraformaldehyde for 24 h, dehydrated, embedded in conventional paraffin and cut into serial sections (5 μm). The sections were stained with hematoxylin and eosin, and histopathologically examined with light microscopy. For TUNEL analysis, the sections were stained with a commercial TUNEL kit purchased from Roche (fluorescein, Basel, Switzerland, cat. no 11684817910). The TUNEL positive nuclei displayed green fluorescence, and all nuclei in the spleen tissues displayed blue fluorescence under a fluorescence microscope. The computational formula for apoptotic index = number of TUNEL positive nuclei/total number of nuclei × 100%. We selected five sections from each group for statistical analysis, and images were taken at a high magnification field (400×) under a fluorescence microscope.

### Redox parameters and inflammatory cytokine analysis

Spleen tissues (approximately 0.5 g) were homogenized in a ninefold volume of precooled normal saline with an Ultra-Turrax homogenizer. The homogenates were centrifuged at 3,000×*g* for 15 min at 4 °C, and the resultant supernatants were collected for protein assays and subsequent measurements.

The activity of several antioxidant enzymes and the levels of oxidative stress indicators were measured with commercial kits. The activity of GPX, TXNRD, and total nitric oxide synthase (TNOS) and the hydroxyl radical inhibition capacity (HRIC) were measured with specific assay kits from the Nanjing Jiancheng Bioengineering Institute of China. The activity of GPX was measured according to the decrease in reduced glutathione absorbance with H_2_O_2_ as the substrate. The activity of TXNRD was measured by the reduced nicotinamide adenine dinucleotide phosphate-dependent reduction of 5,5-dithiobis-(2-nitrobenzoic acid) method. The activity of TNOS was measured with L-Arginine and oxygen substrates. The HRIC was measured by using Fenton reaction. The activity of catalase (CAT) and superoxide dismutase (SOD), and the concentrations of hydrogen peroxide (H_2_O_2_) and malondialdehyde (MDA), were measured with specific assay kits from the Beyotime Institute of Biotechnology of China. The detailed steps were performed according to the manufacturer’s instructions. CAT catalyzes the oxidation of H_2_O_2_ to H_2_O and O_2_. When H_2_O_2_ is in excess, the surplus H_2_O_2_ can oxidize the kit’s chromogenic substrate to form a red product (N-(4-antipyryl)-3-chloro-5-sulfonate-p-benzoquinonemonoimine) via peroxidase. The activity of CAT was determined by measuring the absorbance at the maximum absorption wavelength (520 nm) of the red product. The assay of SOD was based on the reduction of nitroblue tetrazolium (WST-8) to water-insoluble blue formazan. The determination of H_2_O_2_ depends on ferrous iron can be oxidized to ferric iron by H_2_O_2_, and then react with xylenol orange in a specific solution to synthetizing purple products. So the concentration of the H_2_O_2_ can be detected by microplate reader. MDA was estimated by the thiobarbituric acid (TBA) method.

Inflammatory cytokines, comprising IL-1β, IL-6, IL-8, IL-12, IL-10, IL-13, IL-17, tumor necrosis factor-alpha (TNF-α), transforming growth factor beta (TGF-β), NF-κB, NF-κB p65, HIF-1α, and cyclooxygenase (COX-2), were measured with commercial enzyme-linked immunosorbent assay (ELISA) kits (Shanghai Enzyme-linked Biotechnology Co., Ltd., Shanghai, China) according to the manufacturer’s instructions. The intra-assay coefficients of variation for cytokines were less than 10%, and the inter-assay coefficients of variation were less than 15%.

All redox and inflammatory parameters were normalized to protein concentrations, which were measured with a bicinchoninic acid (BCA) protein assay kit (Thermo Fisher Scientific, San Jose, CA, USA).

### Real-time PCR and western blot analysis

The real-time PCR analyses of spleen tissue were conducted as previously described [27]. The primers for selenoprotein genes, the inflammation- and apoptosis-related genes, and the housekeeping gene glyceraldehyde 3-phosphate dehydrogenase (GAPDH) are presented in Supplemental Table 2. The 2^−∆∆Ct^ method was used for quantification with GAPDH as the reference gene, and the relative abundance was normalized to that of the Se-A group.

Spleen tissues were homogenized and lysed with RIPA lysis buffer (50 mmol/L Tris-HCl (pH 7.4), 150 mmol/L NaCl, 1% NP-40 and 0.1% SDS) supplemented with proteinase inhibitors. The total protein concentration was calculated with a BCA Protein Assay Kit (Thermo Fisher Scientific). Equal amounts of protein (30 μg protein/line) were loaded and separated on a 15% sodium dodecyl sulfate–polyacrylamide gel electrophoresis gel. After electrophoresis, the proteins were transferred to a polyvinylidene difluoride membrane, blocked in 5% (w/v) non-fat milk and incubated with the primary antibodies overnight at 4 °C. Primary antibodies against GPX4 (LS-C407839, LifeSpan BioSciences, Seattle, WA, USA), SELENOO (ab172957, Abcam, Cambridge, UK), and GAPDH (2118, Cell Signaling Technology, Danvers, MA, USA) were used. After incubation with the primary antibodies, the membrane was washed in Tris-buffered saline-Tween and then incubated with horseradish peroxidase-conjugated secondary antibody for another 2 h at room temperature. Immuno-reactive bands were visualized with an enhanced chemiluminescence detection kit (Thermo Fisher Scientific). ImageJ software (NIH, Bethesda, MD, USA) was used to measure the blot signal and density.

### Statistical analysis

Statistical differences in the data were assessed with SPSS 22.0 software (SPSS Inc., Chicago, IL, USA). Data were tested for normality with the Shapiro–Wilk test. According to the normality of the data, parametric (two-sided Student’s *t*-test) or non-parametric (Mann–Whitney U test) analysis was used for statistical evaluation of the results. The thresholds for significant differences were *P < 0.05 and ** *P* < 0.01. All figures were prepared with GraphPad Prism 7.0 software (GraphPad Software Inc., La Jolla, CA, USA).

## Results

### Se deficiency alters erythroid parameters and makes erythrocytes osmotically fragile

**Fig. 1:**
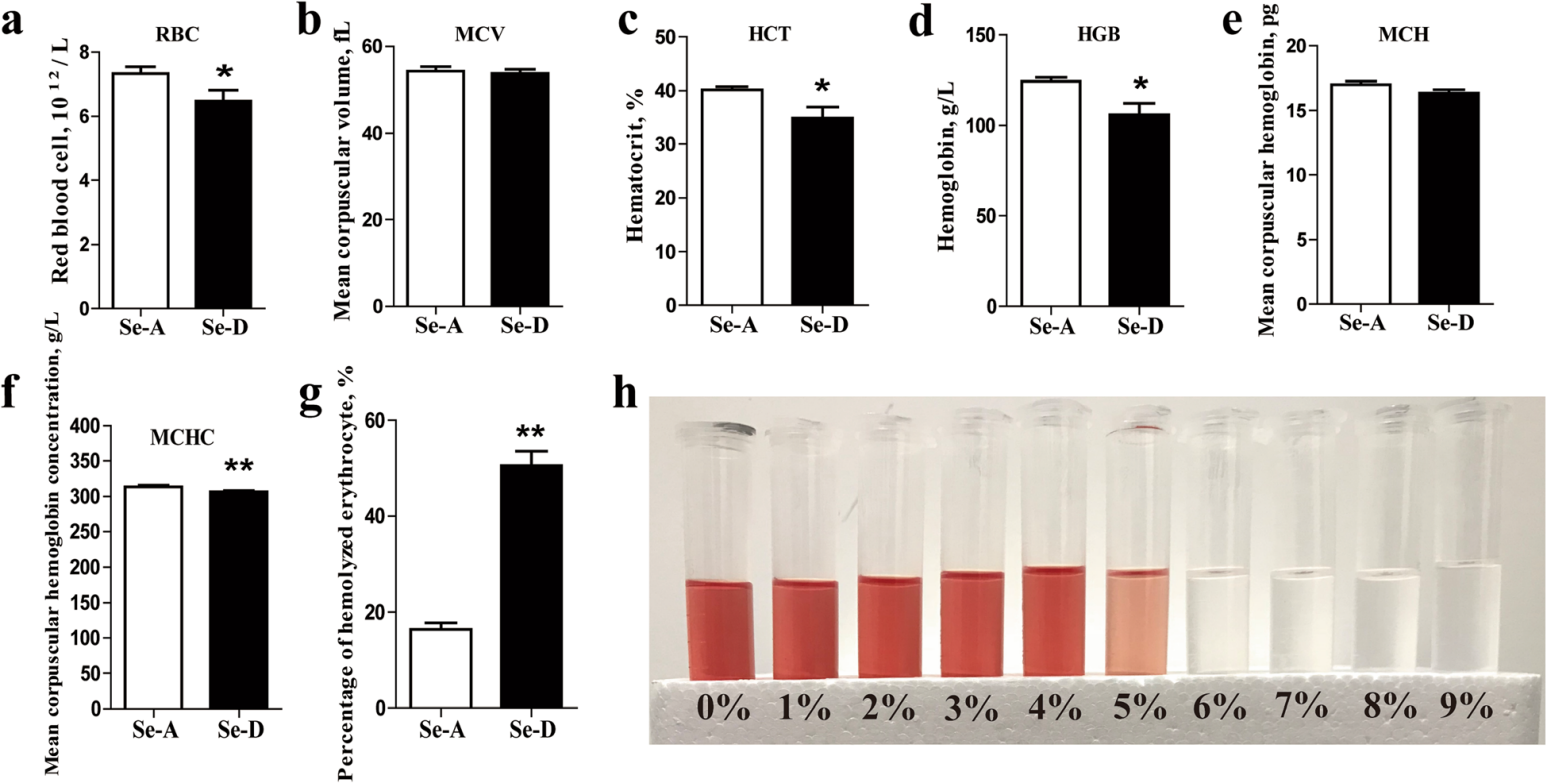
The erythroid parameters of RBC, MCV, HCT, HGB, MCH, and MCHC, and osmotically fragile erythrocytes in the Se-adequate group and Se-deficient group. Values are means ± SEM, *n* = 12. * Different from Se-A, *P* < 0.05; ** different from Se-A, *P* < 0.01

The various erythroid parameters of the Se-A and Se-D groups are shown in Fig. 1a–f. Se deficiency led to significantly lower RBC, HCT, HGB, and MCHC (*P* < 0.05) than the levels in the Se-A group. The RBC, HCT, HGB, and MCHC in the Se-D group were 11.7%, 13.3%, 14.7%, and 3.0% lower, respectively, than those in the Se-A group. The two groups did not differ in MCV and MCH. The hemolysis of porcine erythrocytes in the Se deficient group under different concentrations of buffered salt solution is shown in Fig. 1h. When the concentration of NaCl was 0.5%, the erythrocytes began to hemolyze. Therefore, the hemolysis of the two groups of erythrocytes in 0.5% NaCl solution was selected to evaluate the osmotic fragility of erythrocytes. The Se-D group was significantly more prone to hemolysis (*P* < 0.05) than the Se-A group under hypotonic stress (Fig. 1g). The percentage of hemolyzed of erythrocytes in the Se-D group was 3.1 times that in the Se-A group.

### Effects of se deficiency on histology, se concentration and spleen index

**Fig. 2:**
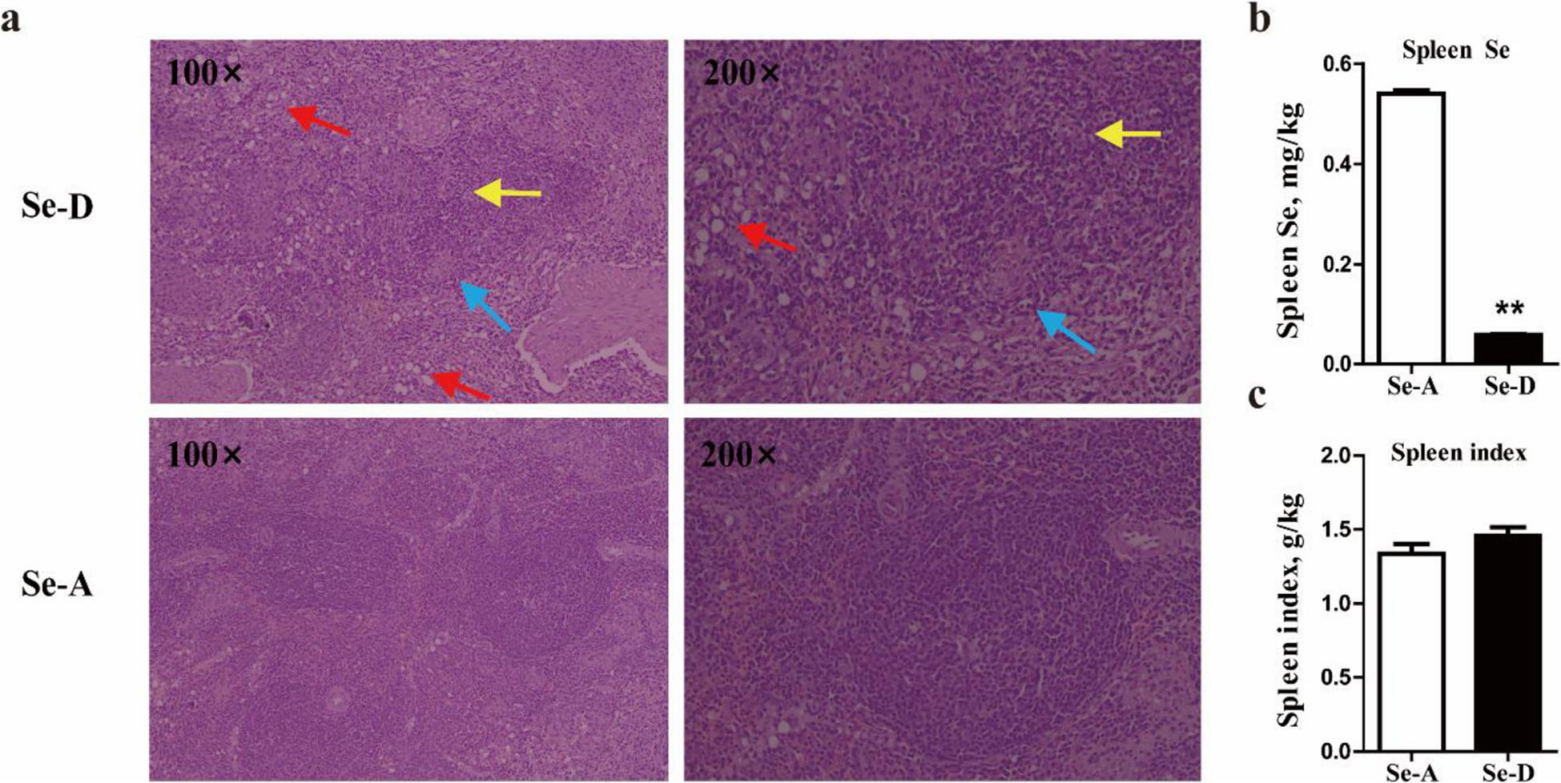
Hematoxylin and eosin staining, Se concentrations, and spleen index in the Se-adequate group and Se-deficient group spleens. Se deficiency caused blurring of the boundary between red and white pulp, and decreased the white pulp volume and cell density (yellow arrow) in the spleen, particularly narrowing of the periarterial lymphatic sheath (blue arrow). In addition, Se deficiency significantly decreased the density and number of splenic cord cells (red arrow), thereby increasing the splenic sinusoid space and the splenic red pulp volume. Photomicrographs are shown at 100× and 200× magnification

Histological staining showed that the red and white pulp of the spleen in the Se-A group had clear structures and clear boundaries (Fig. 2a). The white pulp contained abundant lymphocytes and macrophages, and the cell structure was intact. Se deficiency caused blurring of the boundary between the red and white pulp, and decreased the white pulp volume and cell density (yellow arrow, Fig. 2a) in the spleen, particularly narrowing of the periarterial lymphatic sheath (blue arrow, Fig. 2a). In addition, Se deficiency decreased the density and number of splenic cord cells (red arrow, Fig. 2a), thus increasing the splenic sinusoid space and the splenic red pulp volume. The spleen Se concentration was significantly influenced by dietary Se deficiency (Fig. 2b). The concentrations of Se in spleen tissues in the Se-A group were 10.2 times those in the Se-D group (*P* < 0.001). The spleen index in two group showed no significant difference (Fig. 2c).

### Effects of se deficiency on spleen selenoprotein expression

**Fig. 3:**
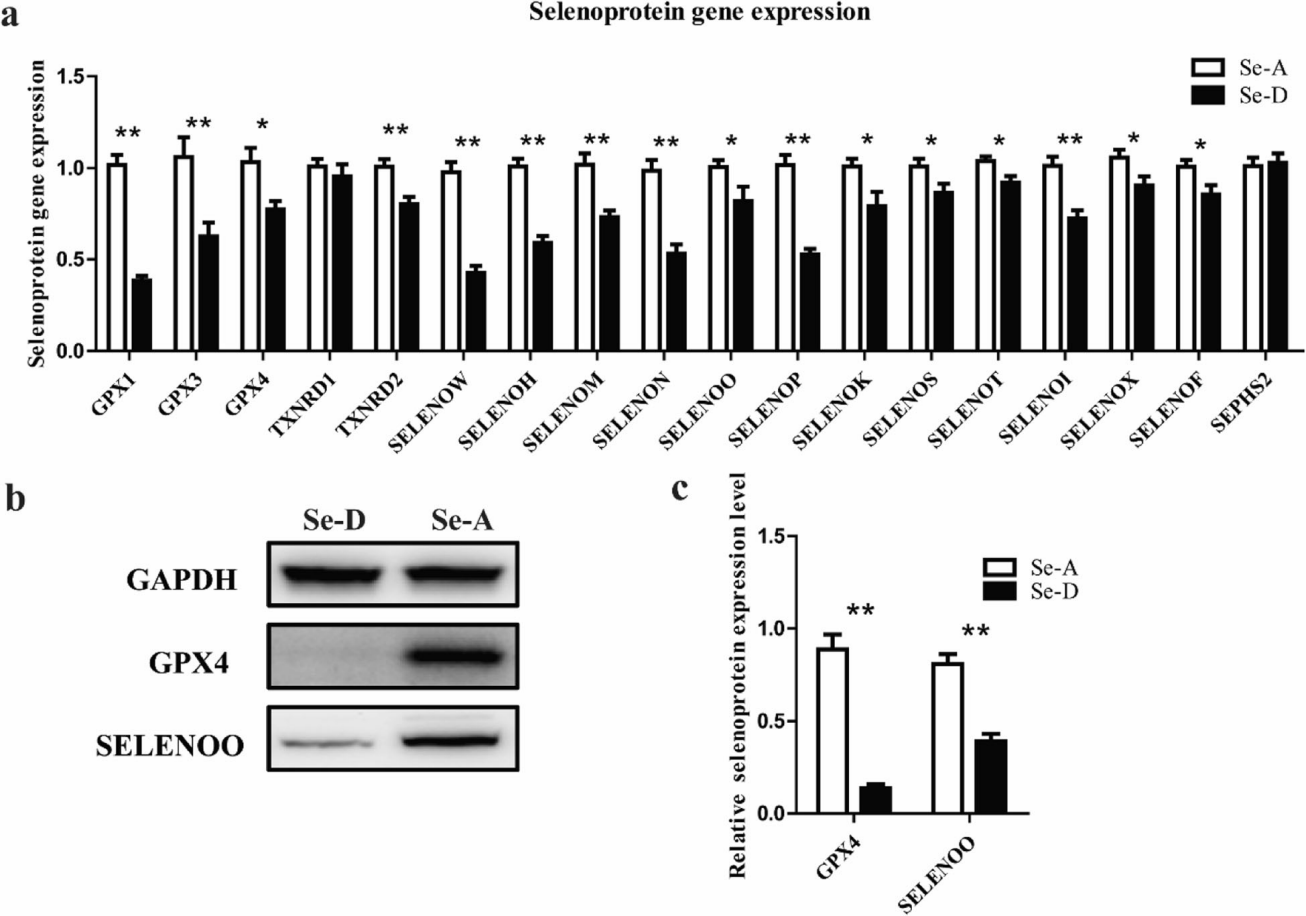
Relative mRNA and protein levels of selenoproteins in the spleen in the Se-adequate group and Se-deficient group. Values are means ± SEM, *n* = 12. * Different from Se-A, *P* < 0.05; ** different from Se-A, *P* < 0.01

The effects of selenium deficiency on the expression of selenoproteins in porcine spleen are shown in Fig. 3. As compared with that in the Se-A group, the expression of 16 of 18 selenoprotein genes was significantly altered under Se deficiency (*P* < 0.05). The relative mRNA levels of selenoprotein (*SELENO*) *T*, *SELENOX*, *SELENOS*, *SELENOF*, and *SELENOO* in the Se-D group were 11.2%, 14.4%, 14.4%, 15.1%, and 18.4% lower, respectively, than those in the Se-A group (*P* < 0.05). The transcript abundance of *TXNRD2*, *SELENOK*, *GPX4*, *SELENOM*, and *SELENOI* in the Se-D group was 20.5%, 21.5%, 25.2%, 28.3%, and 28.6% lower, respectively, than that in the Se-A group (*P* < 0.05). The relative mRNA levels of *GPX3*, *SELENOH*, *SELENON*, *SELENOP*, *SELENOW*, and *GPX1* in the Se-D group were 41.0%, 41.4%, 46.1%, 48.2%, 56.5%, and 62.0% lower, respectively, than those in the Se-A group (*P* < 0.05). In addition, no significant differences (*P* > 0.05) were observed in the relative mRNA levels of *TXNRD1* and *SEPHS2* in the Se-A and Se-D groups. Western blotting (Fig. 3b and 3c) indicated that the protein expression of GPX4 and SELENOO in the Se-D group was 80.7% and 51.8% lower, respectively, than that in the Se-A group (*P* < 0.05).

### Se deficiency causes a redox imbalance in the spleen

**Fig. 4:**
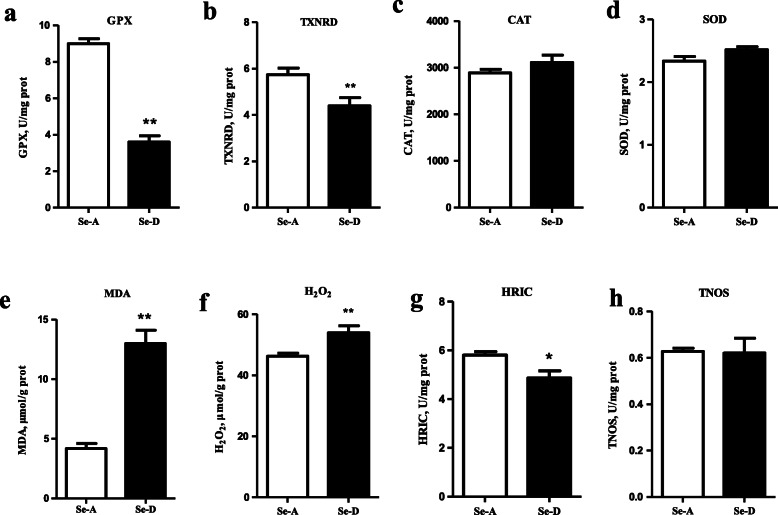
The activity of GPX, TXNRD, CAT, SOD, and TNOS; the hydroxyl radical inhibition capacity; and the content of MDA and H_2_O_2_ in the spleen in the Se-adequate group and Se-deficient group. Values are means ± SEM, *n* = 12. * Different from Se-A, *P* < 0.05; ** different from Se-A, *P* < 0.01

Se deficiency induced oxidative stress in the spleen tissue in pigs (Fig. 4). The activity of GPX and TXNRD in the Se-D group was 59.9% and 23.5% lower, respectively, than that in the Se-A group (*P* < 0.05). The HRIC in the Se-D group was 16.2% (*P* < 0.05) lower than that in the Se-A group. The content of MDA and H_2_O_2_ in the Se-D group was 2.1 times (*P* < 0.05) and 16.6% (*P* < 0.05) greater, respectively, than that in the Se-A group. The activity of SOD, CAT and TNOS in the spleen of pigs did not differ between groups (*P* > 0.05).

### Se deficiency induces spleen inflammation

**Fig. 5:**
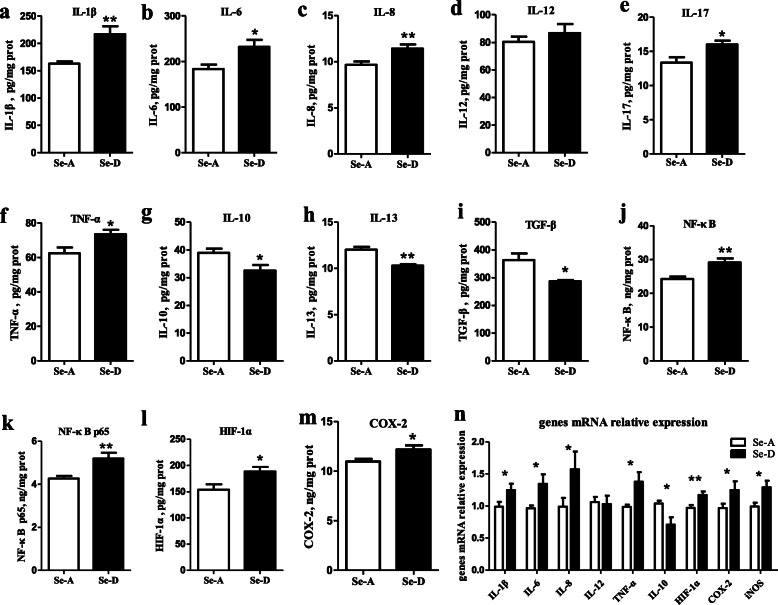
The content of the pro-inflammatory cytokines IL-1β, IL-6, IL-8, IL-12, IL-17, and TNF-α; anti-inflammatory cytokines IL-10, IL-13, and TGF-β; transcription factors NF-κB and HIF-1α; NF-κB p65 and COX-2; and the relative mRNA levels of inflammatory genes in the spleen in the Se-adequate group and Se-deficient group. Values are means ± SEM, *n* = 12. * Different from Se-A, *P* < 0.05; ** different from Se-A, *P* < 0.01

The ELISA results showed that Se deficiency caused inflammation of the spleen tissue. The content of the pro-inflammatory cytokines IL-1β, IL-6, IL-8, IL-17, and TNF-α in the Se-D group was 33.2%, 26.2%, 18.3%, 20.2%, and 17.7% greater, respectively, than that in the Se-A group (*P* < 0.05) (Fig. 5a–f). The content of the pro-inflammatory cytokine IL-12 did not significantly differ between groups (*P* > 0.05) (Fig. 5d). The content of the anti-inflammatory cytokines IL-10, IL-13, and TGF-β in the Se-D group was 16.4%, 14.4%, and 21.0% lower, respectively, than that in the Se-A group (*P* < 0.05) (Fig. 5g–5i). The content of NF-κB, NF-κB p65, HIF-1α, and COX-2 in the Se-D group was 20.1%, 21.9%, 22.5%, and 11.2% (*P* < 0.05) greater than that in the Se-A group (Fig. 5j–m). The effects of Se deficiency on the relative expression of inflammatory cytokine genes in the spleen was consistent with the ELISA results for inflammatory cytokine expression. The relative mRNA levels of *IL-1β*, *IL-6*, *IL-8*, and *TNF-α* in the Se-D group were 26.4–59.2% greater, respectively, than those in the Se-A group (*P* < 0.05). The relative mRNA levels of *IL-10* in the Se-D group were 31.4% lower, respectively, than those in the Se-A group (*P* < 0.05). The transcript abundance of *HIF-1α*, *COX-2*, and *iNOS* in the Se-D group was 20.7%, 29.0%, and 30.1% greater, respectively, than that in the Se-A group (*P* < 0.05).

### Se deficiency induces spleen apoptosis

**Fig. 6:**
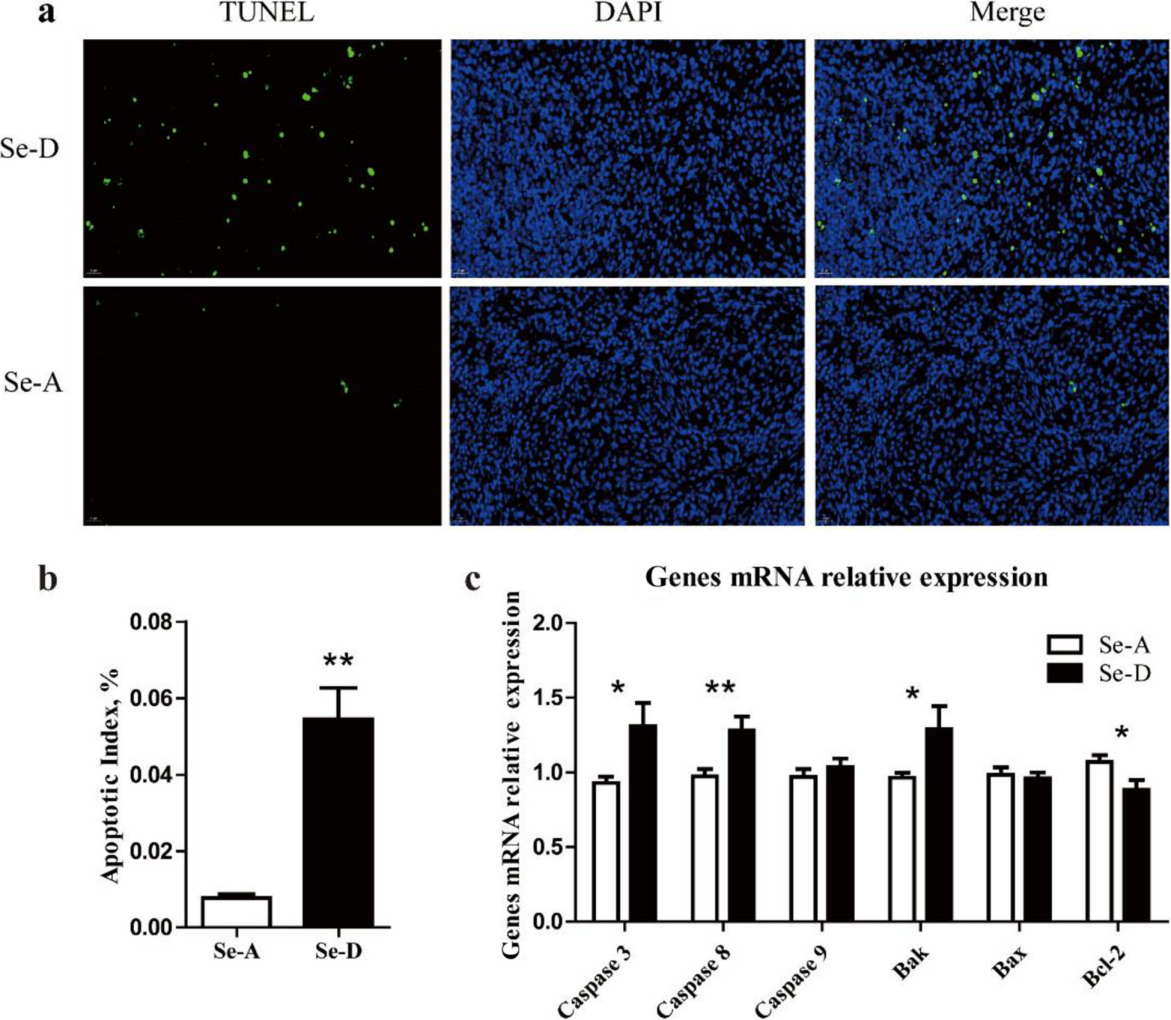
TUNEL staining of spleen tissue and relative mRNA levels of apoptosis genes in the spleen in the Se-adequate group and Se-deficient group. Values are means ± SEM, *n* = 12. * Different from Se-A, *P* < 0.05; ** different from Se-A, *P* < 0.01

The results of TUNEL staining showed that the number of positive nuclei in the Se-D group was markedly higher than that in the Se-A group (*P* < 0.05) (Fig. 6a–b). The apoptotic index of the Se-D group was 6.1 times greater than that in the Se-A group. To further reveal the effect of Se deficiency on apoptosis in the spleen in pigs, we detected the mRNA expression of six apoptosis regulatory genes (Fig. 6c). The relative mRNA levels of *Caspase 3*, *Caspase 8*, and *Bak*, in the Se-D group were 40.3%, 31.3%, and 33.4% greater, respectively, than those in the Se-A group (*P* < 0.05). The transcript abundance of *Bcl-2* in the Se-D group was 17.5% lower, respectively, than that in the Se-A group (*P* < 0.05). There were no significant differences (*P* > 0.05) in the relative mRNA levels of *Caspase 9* and *Bax* in the Se-A and Se-D groups.

## Discussion

The spleen, the largest peripheral lymphoid organ in the body, participates in the immune response, generation of lymphocytes, elimination of aging erythrocytes and storage of blood. Many studies have shown that Se can alleviate the spleen damage caused by mycotoxins and heavy metal elements [28, 29]. However, the mechanism of the effect of Se deficiency on the spleen is not fully understood. Se deficiency leads to a series of changes in the erythrocytes and spleen in pigs, including a decrease in erythrocyte counts, HGB levels and HCT, all of which are pathological signs of perturbed erythropoiesis, similar to previous reports [30]. In our study, the RBC, HCT and HGB in the Se-D group were 11.7%, 13.3%, and 14.7% lower (*P* < 0.05), respectively, than those in the Se-A group, which was basically consistent with the changes in mice caused by Se deficiency [30]. The hemoglobin in erythrocytes is responsible for carrying and transporting oxygen and maintaining normal tissue oxygenation [31]. HGB, MCH, and MCHC together reflect the level of hemoglobin in erythrocytes. The synthesis and accumulation of hemoglobin are characteristics of erythrocyte maturation. In addition, erythrocyte immaturity leads to a decrease in HCT [32]. Together, these findings indicate that Se deficiency increases the proportion of immature erythrocytes. Osmotic fragility tests revealed that the erythrocytes from the Se-D group were more prone to hemolysis than those in Se-A group under hypotonic stress, possibly because of oxidative stress-mediated changes in erythrocyte membranes [33, 34]. The results are similar to those from previous studies on mice and chickens fed a Se-deficient diet [30, 34, 35]. Se deficiency did not significantly affect the spleen index in pigs, in agreement with results obtained in chickens with Se deficiency for 3 weeks [21]. Histological analysis showed that dietary Se deficiency led to splenic injury, as evidenced by a diminished number of lymphocytes and a decreased white pulp region. These changes in spleen tissue probably decreased the cellular immunity of the body. Extensive studies have shown that Se deficiency affects the histological structure of chicken spleen [3, 4], in agreement with our results.

Se deficiency significantly affected Se concentrations and the expression of selenoproteins in the spleen; these effects are likely the primary reasons behind the pathological phenotype observed in this study. The Se concentration in the spleen in the Se-D group was approximately one-tenth that in the Se-A group. The difference in spleen Se concentrations between the two groups was larger than that reported in the kidney [36], and may explain the lower selenoprotein gene expression observed in the spleen. Of the 18 selenoprotein genes examined, only *TXNRD1* and *SEPHS2* did not show decreased mRNA expression under Se deficiency. The splenic GPX4 and SELENOO protein expression decreased significantly in the Se-D group, to a greater extent than the decrease in mRNA expression. The expression of 11 selenoprotein mRNAs in the spleen in pigs fed a Se deficient diet was consistent with findings from previous studies, but the expression of seven selenoprotein mRNAs was not consistent [37]. This difference may be related to the fact that selenoproteins can be regulated at the transcription, translational and post-translational levels. The down-regulated selenoproteins GPX1, GPX3, GPX4, TXNRD2, and SELENOX are well-known redox-active selenoenzymes with important roles in antioxidative stress [38]. The splenic activity of GPX and TXNRD decreased significantly in the Se-D group, which was positively correlated well with their mRNA abundance. SELENOP, a major Se-containing protein in the plasma, plays a pivotal role in Se transport and antioxidative defense [39]. SELENOW and SELENOH have anti-oxidation and anti-apoptosis functions [40, 41]. Se deficiency significantly decreases the mRNA expression of six endoplasmic reticulum (ER) resident selenoprotein genes (*SELENOM*, *SELENON*, *SELENOT*, *SELENOF*, *SELENOK*, and *SELENOS*) in the spleen. ER-resident selenoproteins are thought to have oxidoreductase activity and have been implicated in a range of processes including ER stress, inflammation, Ca^2+^ homeostasis and muscle development [42]. SELENOS regulates oxidative stress, and decreased *SELENOS* expression appears to specifically exacerbate the inflammatory and apoptotic profiles [43, 44]. SELENOT exerts protective effects in acute kidney injury through suppression of oxidative stress and apoptosis [45].

The low expression of selenoproteins in the Se-D group induced a series of reactions, including redox imbalance, inflammation and apoptosis. The enzymatic activity of CAT and SOD in the Se-D group did not decrease, but the decrease in the GPX and TXNRD activity of the selenoenzyme antioxidant system resulted in perturbation of the redox homeostasis and ROS elevation. MDA, a lipid peroxidation indicator of oxidative stress, significantly increased in the Se-D group, possibly because of excessive downregulation of GPX4 protein production. GPX4 is the only GPX enzyme that breaks down phospholipid hydroperoxides [46], and its deficiency leads to lipid peroxidation. The content of H_2_O_2_, the principal ROS, increased significantly in the Se-D group, thereby indicating that the spleen was in a state of oxidative stress after Se deficiency. Se deficiency decreased the hydroxyl radical inhibition, a result reflecting the increase in the levels of highly reactive free oxygen radicals in the spleen. These results suggested that Se deficiency decreased the antioxidant capacity and increased the production of ROS, thus leading to oxidative stress in the spleen, in agreement with previous reports in the spleen and other tissue [47, 48].

High level of oxidative stress and inflammation are inseparably linked and participate in a self-perpetuating vicious circle. In the present study, the levels of pro-inflammatory cytokines (IL-1β, IL-6, IL-8, IL-17, and TNF-α) significantly increased (*P* < 0.05), whereas those of anti-inflammatory cytokines (IL-10, IL-13, and TGF-β) significantly decreased (*P* < 0.05). Simultaneously, the gene expression of inflammatory cytokines showed the similar results. Se deficiency first induces oxidative stress and further activates NF- κB pathway to initiate inflammation. NF-κB is a redox-sensitive transcription factor that regulates expression of proinflammatory cytokines and chemokines, and thus mediates inflammatory responses [49]. The increased expression of COX-2 and iNOS, important downstream regulatory genes in the NF-κB signaling pathway, promoted inflammation in the Se deficient spleen, similarly to the results of other studies [50, 51]. HIF is a transcription factor that not only responds to hypoxia but also is activated by some non-hypoxic stimuli, including bacterial lipopolysaccharide, ROS, TNF-α, and IL-18 [52, 53]. HIF-1α plays an important role in metabolic disorders and inflammation [54]. High glucose increases HIF-1α expression, and subsequently promotes inflammation and fibrosis in rat glomerular mesangial cells [55]. HIF-1α is also essential for myeloid cell-mediated inflammation [56]. In our research, oxidative stress induced by Se deficiency promoted the expression of HIF-1α and its downstream inflammation factors (inflammatory cytokines and TGF-β).

At lower doses, ROS have been associated with induction of cell survival responses, whereas higher doses activate death processes such as apoptosis [17]. In the present study, TUNEL staining showed that splenocyte apoptosis was enhanced in the Se-D group. In addition, Se deficiency upregulated the expression of Caspase 3, Caspase 9, and Bak, but downregulated that of Bcl-2 in the spleen in pigs, thus revealing that Se deficiency induces splenocyte apoptosis through the mitochondrial pathway. The mitochondrial pathway of apoptosis is regulated by the caspase family, the Bcl-2 family, apoptosis inducing factor, and second mitochondria-derived activator of caspases [57]. Normally, Bcl-2 and caspase are mutually restricted to maintain a balance between cell proliferation and death. If this balance is disturbed, apoptosis occurs. Previous studies have shown that Se deficiency affects immune organs, cardiomyocytes and duodenal villus cell apoptosis [22, 58, 59]. Oxidative stress and low expression of selenoproteins induced by Se deficiency may be the cause of splenocyte apoptosis. The decrease in the density and number of white pulp cells and splenic cord cells shown by histological staining may be the result of splenocyte apoptosis. We also speculate that the low expression of GPX4 induced by Se deficiency may lead to cell ferroptosis. We determined the expression of five ferroptosis related genes, and the results showed that Se deficiency decreased the expression of *SLC7A11* and *FSP1*, but the expression of *ALOX5*, *ACSL4*, and *SLC3A2* did not change (Supplemental Fig. 1). The relationship between Se deficiency and ferroptosis needs to be further explored, which is what we are planning to do in the future.

## Conclusions

Herein, we used a pig model to reveal the damage to the erythrocytes and spleen caused by dietary Se deficiency, as evidenced by changes in erythroid parameters, the hemolysis ratio, histopathologic lesions, and the apoptosis index. Se deficiency decreased the expression of most selenoproteins in the spleen, thus weakening the antioxidation ability, and resulting in ROS accumulation and oxidative stress. Moreover, the Se deficiency-induced redox imbalance induced inflammation by activating the NF-κB and HIF-1α pathways, and stimulated apoptosis through regulated the caspase family and the Bcl-2 family. We conclude that the spleen pathological damage caused by Se deficiency is closely associated with oxidative stress, inflammation, and apoptosis.

## Supplementary Information

**Additional file 1: Supplemental Table 1**. Ingredient and nutrient composition of the basal diet

**Additional file 2: Supplemental Table 2**. List of primers used for real-time PCR analysis

**Additional file 3: Figure S1** Relative mRNA levels of ferroptosis related genes in the spleen in the Se-adequate group and Se-deficient group

## Data Availability

The data were shown in the main manuscript and supplemental materials.

## Abbreviations

Se: Selenium
Bak: BCL2 antagonist killer 1
Bax: BCL-2-associated X protein
BCA: Bicinchoninic acid
Bcl-2: B-cell lymphoma 2
Caspase 3: Cysteinyl aspartate specific proteinase 3
Caspase 8: Cysteinyl aspartate specific proteinase 8
Caspase 9: Cysteinyl aspartate specific proteinase 9
CAT: Catalase
COX-2: Cyclooxygenase
ELISA: Enzyme-linked immunosorbent assay
GAPDH: Glyceraldehyde 3-phosphate dehydrogenase
GPX: Glutathione peroxidase
H_2_O_2_: Hydrogen peroxide
HCT: Hematocrit
HGB: Hemoglobin
HIF-1α: Hypoxia inducible factor 1 alpha
HRIC: Hydroxyl radical inhibition capacity
IL: Interleukin
iNOS: Inducible nitric oxide synthase
MCH: Mean corpuscular hemoglobin
MCHC: Mean corpuscular hemoglobin concentration
MCV: Mean corpuscular volume
MDA: Malondialdehyde
NF-κB: Nuclear factor kappa B
Nrf2: Nuclear erythroid-2 related factors
RBC: Red blood cell
ROS: Reactive oxygen species
SELENOF: Selenoprotein F
SELENOH: Selenoprotein H
SELENOI: Selenoprotein I
SELENOK: Selenoprotein K
SELENOM: Selenoprotein M
SELENON: Selenoprotein N
SELENOO: Selenoprotein O
SELENOP: Selenoprotein P
SELENOS: Selenoprotein S
SELENOT: Selenoprotein T
SELENOW: Selenoprotein W
SELENOX: Selenoprotein X
SeMet: Selenomethionine
SEPHS2: Selenophosphate synthetase 2
SOD: Superoxide dismutase
TGF-β: Transforming growth factor beta
TNF-α: Tumor necrosis factor alpha
TNOS: Total nitric oxide synthase
TUNEL: Terminal deoxynucleotidyl transferase nick-end labeling
TXNRD: Thioredoxin reductase
