# Supplementary material for: Selenium deficiency induces spleen pathological changes in pigs by decreasing selenoprotein expression, evoking oxidative stress, and activating inflammation and apoptosis
Source: J Anim Sci Biotechnol. 2021 May 17;12:65. doi: 10.1186/s40104-021-00587-x (PMC8127211; doi:10.1186/s40104-021-00587-x)
Supplement: Supplementary file 1 — Additional file 1: Supplemental Table 1. Ingredient and nutrient composition of the basal diet [file 40104_2021_587_MOESM1_ESM.docx]

Supplemental Table 1. Ingredient and nutrient composition of the basal diet for pigs

| Item | Starter (weight＜25 kg^a^) | Grower (weight＞25 kg^b^) |
| --- | --- | --- |
| Ingredients |  |  |
| Maize, % | 66 | 74.7 |
| Soybean meal, % | 26.2 | 20 |
| Soybean oil, % | 3.6 | 1.6 |
| Sodium chloride, % | 0.5 | 0.3 |
| Limestone, % | 0.9 | 0.9 |
| Dicalcium phosphate, % | 1 | 0.9 |
| Lysine, % | 0.8 | 0.6 |
| Premix ^1^, % | 1 | 1 |
| Digestible energy, Kcal /kg | 3493.28 | 3405.15 |
| Crude protein, % | 15.88 | 14.17 |
| Calcium, % | 0.71 | 0.67 |
| available phosphorus, % | 0.29 | 0.27 |
| Lysine, % | 1.23 | 0.98 |
| Methionine, % | 0.36 | 0.28 |
| Threonine, % | 0.73 | 0.59 |
| Tryptophan, % | 0.20 | 0.17 |

^1^ Premix provided (per kg feed): Cu (5 mg ^a^, 4 mg ^b^), I (0.14 mg ^a, b^), Fe (100 mg ^a^, 60 mg ^b^), Mn (3 mg ^a^, 2 mg ^b^), Zn (80 mg ^a^, 60 mg ^b^), vitamin A (1750 IU ^a^, 1300 IU ^b^), vitamin D_3_ (200 IU ^a^, 150 IU ^b^), vitamin E (11 IU ^a, b^), vitamin K_3_ (0.5 mg ^a, b^), biotin (0.05 mg ^a, b^), choline (0.4 g ^a^, 0.3 g ^b^), folic acid (0.3 mg ^a, b^), niacin (30 mg ^a, b^), d-pantothenic acid (9 mg ^a^, 8 mg ^b^), vitamin B_1_ (1 mg ^a, b^), vitamin B_2_ (3 mg ^a^, 2.5 mg ^b^), vitamin B_6_ (3 mg ^a^, 1 mg ^b^), vitamin B_12_ (15 μg ^a^, 10 μg ^b^).
