# Supplementary material for: Selenium deficiency induces spleen pathological changes in pigs by decreasing selenoprotein expression, evoking oxidative stress, and activating inflammation and apoptosis
Source: J Anim Sci Biotechnol. 2021 May 17;12:65. doi: 10.1186/s40104-021-00587-x (PMC8127211; doi:10.1186/s40104-021-00587-x)
Supplement: Supplementary file 2 — Additional file 2: Supplemental Table 2. List of primers used for real-time PCR analysis [file 40104_2021_587_MOESM2_ESM.docx]

Supplemental Table 2. List of primers used for selenoprotein, inflammatory, and apoptosis gene Real-Time PCR analysis

| Gene ^1^ | 5’ to 3’ | Accession NO. |
| --- | --- | --- |
| *GPX1* | Forward: GATGCCACTGCCCTCATGA  Reverse: TCGAAGTTCCATGCGATGTC | AF532927 |
| *GPX3* | Forward: TGCACTGCAGGAAGAGTTTGAA  Reverse: CCGGTTCCTGTTTTCCAAATT | AY368622 |
| *GPX4* | Forward: TGAGGCAAGACGGAGGTAAACT  Reverse: TCCGTAAACCACACTCAGCATATC | NM_214407 |
| *TXNRD1* | Forward: GATTTAACAAGCGGGTCATGGT  Reverse: CAACCTACATTCACACACGTTCCT | AF537300 |
| *TXNRD2* | Forward: TCTTGAAAGGCGGAAAAGAGAT  Reverse: TCGGTCGCCCTCCAGTAG | GU181287 |
| *SELENOF* | Forward: ACAGCCCTGCCAAGCAGAT  Reverse: AACAGGGAGGCTGGGTAACAC | EF178474 |
| *SELENOP* | Forward: AACCAGAAGCGCCAGACACT  Reverse: TGCTGGCATATCTCAGTTCTCAGA | EF113596 |
| *SELENOT* | Forward: GGCTTAATAATCGTTGGCAAAGA  Reverse: TGGCCCCATTGCCAGATA | GQ140624 |
| *SELENOW* | Forward: CACCCCTGTCTCCCTGCAT  Reverse: GAGCAGGATCACCCCAAACA | AF380118 |
| *SELENOS* | Forward: GAGGCAGAGGCACCTGGAT  Reverse: CTGCTAAAGCCTCCTGTCGTTT | AY609646 |
| *SELENON* | Forward: ACCTGGTCCCTGGTGAAAGAG  Reverse: AGGCCAGCCAGCTTCTTGT | EF113595.2 |
| *SELENOK* | Forward: CAGGAAACCCCCCTAGAAGAA  Reverse: CTCATCCACCGGCCATTG | DQ372075 |
| *SELENOH* | Forward: TGGTGGAGGAGCTGAAGAAGTAC  Reverse: CGTCATAAATGCTCCAACATCAC | HM018602 |
| *SELENOI* | Forward: GATGGTGTGGATGGAAAGCAA  Reverse: GCCATGGTCAAAGAGTTCTCCTA | AK347874 |
| *SEPHS2* | Forward: TGGCTTGATGCACACGTTTAA  Reverse: TGCGAGTGTCCCAGAATGC | EF033624 |
| *SELENOO* | Forward: CTTCCGACCCCAGATGGAT  Reverse: GGTTCGACTGTGCCAGCAT | AK236851 |
| *SELENOM* | Forward: CAGCTGAATCGCCTCAAAGAG  Reverse: GAGATGTTTCATGACCAGGTTGTG | FJ968780 |
| *SELENOX* | Forward: ATCCCTAAAGGCCAAGAATCATC  Reverse: GGCCACCAAGCAGTGTTCA | EF113597 |
| *IL-1β* | Forward: CTCTCCAGCCAGTCTTCATTG | NM_214055.1 |
|  | Reverse: GGTCATTATTGTTGTCACCGTAGT |  |
| *IL-6* | Forward: GCTGCAGTCACAGAACGAGT | NM_214399.1 |
|  | Reverse: CAGGTGCCCCAGCTACATTA |  |
| *IL-8* | Forward: AACTGGCTGTTGCCTTCTTGGC | NM_213867.1 |
|  | Reverse: GGGTGGAAAGGTGTGGAATGCG |  |
| *IL-12* | Forward: GAGGCCTGCTTACCACTTGA | NM_213993.1 |
|  | Reverse: GCTAAGGCACAGGGTTGTCA |  |
| *TNF-**α* | Forward: GGCCCAAGGACTCAGATCAT | NM_214022.1 |
|  | Reverse: CTGTCCCTCGGCTTTGACAT |  |
| *IL-10* | Forward: GTGGCAGCCAGCATTAAGTC | NM_214041.1 |
|  | Reverse: AACTCTTCACTGGGCCGAAG |  |
| *HIF-1α* | Forward: CAGCCAGATGATCGTGCAAC | NM_001123124.1 |
|  | Reverse: TCTACAGGCTAAGTCAGAGGGT |  |
| *COX-2* | Forward: TGGTCTGGTGCCTGGTCTGATG | NM_214321.1 |
|  | Reverse: CGTCTGGAACAGCCGTTCATCG |  |
| *iNOS* | Forward: CATCACCACGCCTCCAACTCAG | NM_001143690.1 |
|  | Reverse: AGTCTCAAGCCTCTGCCTCTCG |  |
| *Caspase 3* | Forward: TTGAGACGGACAGTGGGACT | NM_214131.1 |
|  | Reverse: CCGTCCTTTGAATTTCGCCAG |  |
| *Caspase 8* | Forward: CCAGGATTTGCCTCCGGTTA | NM_001031779.2 |
|  | Reverse: TGGGATGTAGTCCAGGCTCA |  |
| *Caspase 9* | Forward: AGCGCACTGGCTCCAATATC | XM_003127618.4 |
|  | Reverse: AGCCTGGACCATTTGCTTGG |  |
| *Bak* | Forward: GACATCAACCGGCGATACGA | XM_021098603.1 |
|  | Reverse: CTGGAGGCGATCTTGGTGAA |  |
| *Bax* | Forward: TCTGAGCAGATCATGAAGACAGG | XM_003127290.5 |
|  | Reverse: TGAGACACTCGCTCAACTTCT |  |
| *Bcl-2* | Forward: AGCATGCGGCCTCTATTTGA | XM_021099593.1 |
|  | Reverse: GGCCCGTGGACTTCACTTAT |  |
| *ALOX5* | Forward: CATGGCCCGATTCCGTAAGA | XM_021072736.1 |
|  | Reverse: ATCCGGTCTGGGGACAAGTA |  |
| *ACSL4* | Forward: GCAGCGCCTCTGATTGAAAG | NM_001038694.1 |
|  | Reverse: CAGCTAGCGAGTCGAAGTGT |  |
| *SLC3A2* | Forward: ACCCCGCTTTCGGTTCTAAG | XM_003353809.4 |
|  | Reverse: CACCTGAGTGGAGTCGAACC |  |
| *SLC7A11* | Forward: CATCGGGACCATCATCGGAG | XM_021101587.1 |
|  | Reverse: CATACGGTCCAGACGACCAG |  |
| *FSP1* | Forward: TGGTGGTGGAGATAGACCTGAA | XM_005657396.3 |
|  | Reverse: GCTGGCTGGAGACCTTGTTAA |  |
| *GAPDH* | Forward: GGAAGAGTGAGTGTCACTGTTGAAGT  Reverse: GAGAAACCTGCAAAATATGATGACAT | AF017079 |

^1^Abbreviation: *GPX*, glutathione peroxidase; *TXNRD*, thioredoxin reductase; *SELENOF*, selenoprotein F; *SELENOP*, selenoprotein P; *SELENOT*, selenoprotein T; *SELENOW*, selenoprotein W; *SELENOS*, selenoprotein S; *SELENON*, selenoprotein N; *SELENOK*, selenoprotein K; *SELENOH*, selenoprotein H; *SELENOI*, selenoprotein I; *SEPHS2*, selenophosphate synthetase 2; *SELENOO*, selenoprotein O; *SELENOM*, selenoprotein M; *SELENOX*, selenoprotein X.; *IL-1β*, interleukin-1β; *IL-6*, interleukin-6; *IL-8*, interleukin-8; *IL-12*, interleukin-12; *TNF-α*, tumor necrosis factor alpha; *IL-10*, interleukin 10; *HIF-1α*, hypoxia inducible factor-1 alpha; *COX-2*, cyclooxygenase; iNOS, inducible nitric oxide synthase; *Caspase*, cysteinyl aspartate specific proteinase; *Bak*, BCL2 antagonist killer 1; *Bax*, BCL-2-associated X protein; *Bcl-2*, B-cell lymphoma 2; *GAPDH*, glyceraldehyde 3-phosphate dehydrogenase; *ALOX5*, arachidonate 5-lipoxygenase; *ACSL4*, acyl-CoA synthetase long chain family member 4; *SLC3A2*, solute carrier family 3 member 2; *SLC7A11*, solute carrier family 7 member 11; *FSP1*, ferroptosis suppressor protein 1
