# Supplementary material for: Selenium deficiency induces spleen pathological changes in pigs by decreasing selenoprotein expression, evoking oxidative stress, and activating inflammation and apoptosis
Source: J Anim Sci Biotechnol. 2021 May 17;12:65. doi: 10.1186/s40104-021-00587-x (PMC8127211; doi:10.1186/s40104-021-00587-x)
Supplement: Supplementary file 3 — Additional file 3: Figure S1 Relative mRNA levels of ferroptosis related genes in the spleen in the Se-adequate group and Se-deficient group [file 40104_2021_587_MOESM3_ESM.docx]

**Figure captions**

**Fig. S1** Relative mRNA levels of ferroptosis related genes in the spleen in the Se-adequate group and Se-deficient group. Values are means ± SEM, n = 12. * Different from Se-A, *P* < 0.05; ** different from Se-A, *P* < 0.01. *ALOX5*, arachidonate 5-lipoxygenase; *ACSL4*, acyl-CoA synthetase long chain family member 4; *SLC3A2*, solute carrier family 3 member 2; *SLC7A11*, solute carrier family 7 member 11; *FSP1*, ferroptosis suppressor protein 1

**Fig. S1**
